# Optimizing the Synergistic Role of Diode Laser and Drug Therapies in the Management of Oral Submucous Fibrosis: A Case Report

**DOI:** 10.7759/cureus.70028

**Published:** 2024-09-23

**Authors:** Siddhartha Varma, Mugdha V Karambelkar, Girish Suragimath, Chandrashekar Yavagal, Sachin B Mangalekar, Sameer A Zope, Rashmi Gangavati

**Affiliations:** 1 Department of Periodontology, School of Dental Sciences, Krishna Vishwa Vidyapeeth (Deemed to be University), Karad, IND; 2 Department of Pedodontics and Preventive Dentistry, Bapuji Dental College and Hospital, Davangere, IND; 3 Department of Periodontology, Bharati Vidyapeeth (Deemed to be University) Dental College and Hospital, Sangli, IND; 4 Department of Oral Pathology and Microbiology, School of Dental Sciences, Krishna Vishwa Vidyapeeth (Deemed to be University), Karad, IND

**Keywords:** areca nut, diode laser therapy, osmf, pbm, smokeless tobacco(st)

## Abstract

Oral submucous fibrosis (OSMF) is one of the most common precancerous conditions of the oral mucosa involving any part of the oral cavity resulting in tissue scarring, dysphagia, and trismus. Habits of chewing areca nut, tobacco, and pan masala and smoking have revealed a strong association with the occurrence of OSMF. Due to its high morbidity and high malignant transformation rate (MTR), continuous efforts have been made to develop effective treatment options. Based on the stage of the disease, various treatment modalities such as medical and surgical therapies are available, each with its own set of advantages and disadvantages. Photobiomodulation (PBM) is an emerging minimally invasive therapy that can be utilized to treat OSMF through its anti-inflammatory and analgesic effects. With the advent of dual-wavelength diode lasers, the current case report focuses on a novel treatment strategy for managing OSMF in combination with pharmacological and oral physiotherapy to achieve optimal results.

## Introduction

Oral submucous fibrosis (OSMF) is a potentially malignant disorder affecting the oral cavity and oropharynx, predominantly observed in Southeast Asian countries and the Indian subcontinent. It is characterized by excessive tissue healing resulting from physical, chemical, or mechanical trauma. Notably, areca nut consumption significantly contributes to the development of oral fibrosis. Arecoline, a component of areca nut, has been shown to elevate levels of reactive oxygen species, upregulate the expression of various cytokines, and activate specific signaling pathways such as mitogen-activated protein kinase kinase (MEK), cyclooxygenase-2 (COX2), and phosphatidylinositol 3-kinase (PI3K), all contributing to fibrosis. However, oral fibrosis can result from other factors such as oral lichen planus, tobacco, misi, tamarind seed, khat, slaked lime-induced alkaline injury, graft versus host disease, and iron deficiency anemia [1]. Pindborg and Sirsat defined the condition as an insidious chronic disease affecting any part of the oral cavity and sometimes the pharynx. Although occasionally preceded by and/or associated with vesicle formation, it is always associated with a juxta epithelial inflammatory reaction, followed by a fibroelastic change of the lamina propria, with epithelial atrophy leading to the stiffness of the oral mucosa and causing trismus and an inability to eat [2].

Countries such as India, Bangladesh, Sri Lanka, Pakistan, Taiwan, China, Kenya, Saudi Arabia, and the United Kingdom; Polynesia; Micronesia; and other regions where Asians have migrated have the highest rates of OSMF. Initially, most patients experience sensitivity to spicy foods and different degrees of rigidity in the lips, tongue, and palate, leading to limited mouth opening and tongue movement [3]. OSMF represents a significant health risk, as it exhibits an overall 6% chance of malignant transformation rate (MTR). This is significantly higher than the reported MTR for other oral potentially malignant disorders such as oral lichen planus (1.4%) and oral lichenoid lesions (3.8%) [4]. As per the World Health Organization (WHO), >5 million people are affected by OSMF globally [5]. It can involve any age group, including school-going children, with more involvement among the young to middle age groups [6,7].

The spurt in cases of OSMF, especially among the youth, is due to commercialization, effective pricing strategies, marketing, the widespread availability of products, and the psychosocial impact of surrounding peer influences. The most common reasons cited by patients regarding the initiation of the habit of consumption of areca nut and its by-products in the form of gutkha or mawa were tension and stress [6,7].

Treatment options for OSMF include conservative methods, medical treatment, and surgery. It is necessary to consider nutritional support, psychological counseling, habit cessation, and pharmacological management before opting for surgery [8]. Minimally invasive therapies can be effective and beneficial, as patient compliance is improved with enhanced comfort during the treatment. Laser photobiomodulation (PBM) is one such minimally invasive therapy for oral submucous fibrosis. It reduces oral mucositis, enhances vascularization, and promotes healing and repair [9]. The current case report focuses on combination therapy, including the nonsurgical and minimally invasive surgical management of OSMF.

## Case presentation

**Figure 1:**
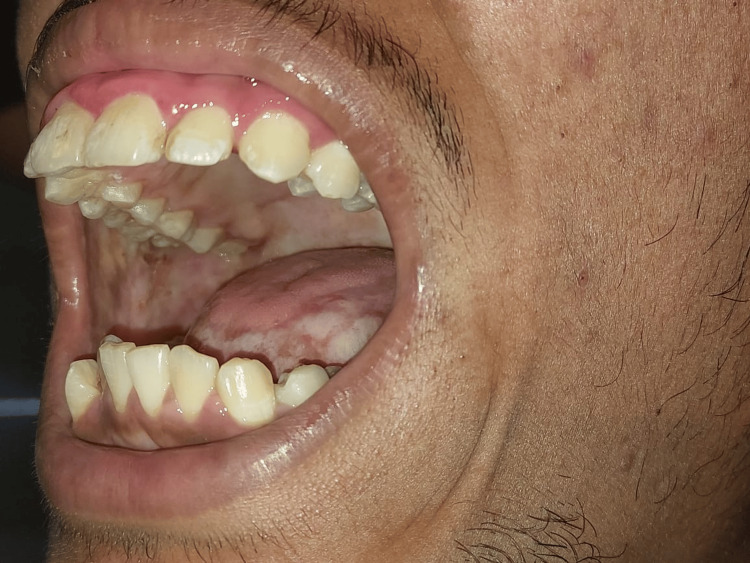
Pale buccal mucosa and palate with fibrous bands involving borders of the tongue

A 22-year-old male presented to the department of periodontology with progressively limited mouth opening, severe burning sensation, and difficulty in speech. The reduced mouth opening resulted in difficulty in maintaining oral hygiene, nutritional deficiency, social anxiety, speech problems, etc. The patient had the habit of chewing "kharra" (a mixture of raw areca nuts mixed with tobacco leaves and slaked lime) seven to eight times per day for the past eight years. The patient reported no history of diabetes, hypertension, drug allergies, or other systemic diseases. The oral examination revealed the whitening and stiffening of the oral mucosa, with the presence of fibrous bands (Figure 1).

**Figure 2:**
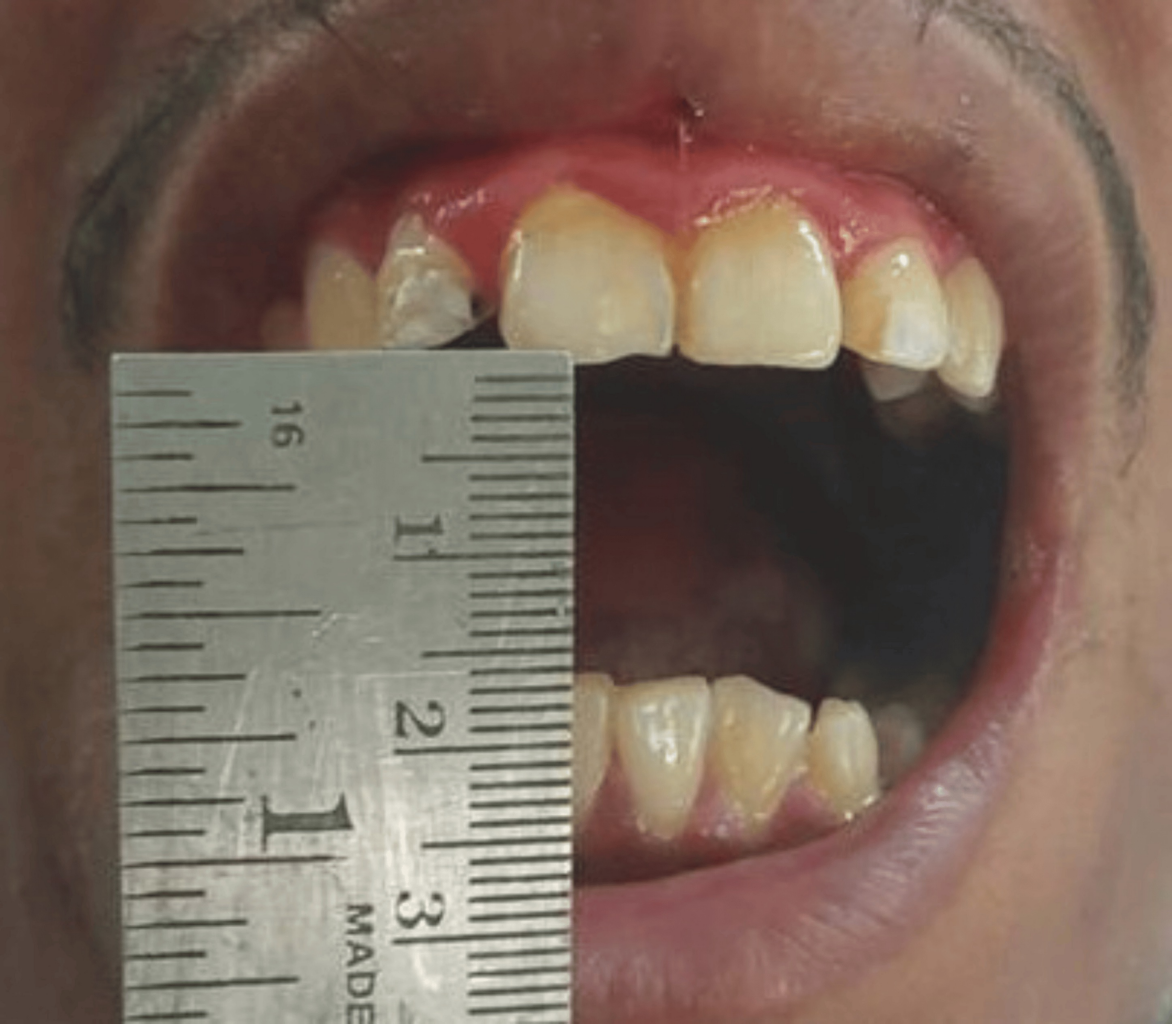
Preoperative inter-incisor distance of 16 mm

The labial mucosa was notably tough, inelastic, and opaque upon palpation, particularly in the molar regions, along with the blanched appearance of the soft palate and pharynx. The inter-incisor distance (IID) was 16 mm (Figure 2), with a sunken cheek appearance.

Vertical bands were palpable in both the right and left buccal mucosa, and circular bands were felt in the maxillary and mandibular labial mucosa. Additionally, a depapillated tongue with restricted tongue movement and a shrunken bud-like uvula was observed, and the patient was reluctant to make eye contact and was uncomfortable during the interaction. Due to the severity of the burning sensation, the patient had to restrict himself to very bland and minimal dietary options. Based on the clinical presentation, the patient was diagnosed with grade III OSMF [10].

Treatment

The patient was educated and motivated to cease the habit and informed about the treatment plan. The remaining treatment commenced after psychiatric consultation at the department of psychiatry of Krishna Institute of Medical Sciences, Karad. Based on the clinical condition, the patient was given the option of surgical and minimally invasive intervention with a laser. The patient was reluctant with the conventional surgical approach and opted for laser therapy. Blood investigations such as complete blood count, blood glucose, and bleeding and clotting time were all within normal limits.

Treatment protocol

**Figure 3:**
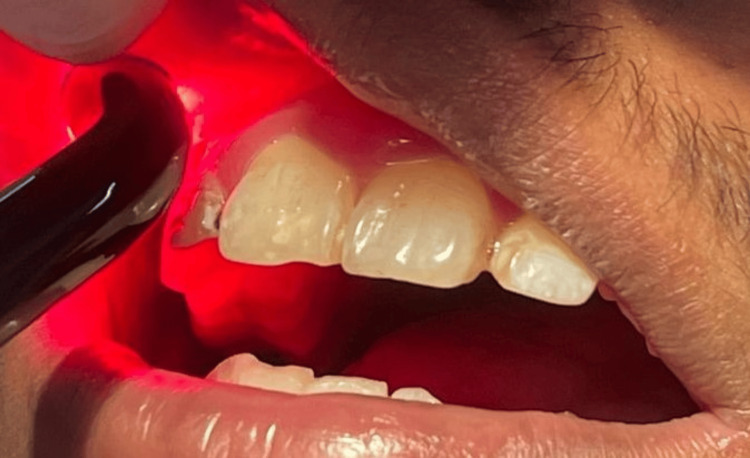
Photobiomodulation in scanning motion with a diode laser (NovoLase Gold 810+640 nm)

The patient was recalled for therapy thrice a week for six weeks, which was done by the following protocol: in the first session of the week, the photobiomodulation (PBM) procedure was carried out using a diode laser (NovoLase Gold, NovoLase Technologies, Hubballi, India) 810 nm, at 100 mW for 60 seconds, delivering 6 J/cm^2^ in a scanning motion through a continuous mode covering the entire mucosa and palate (Figure 3).

In the second session of the week, hyaluronidase 1500 international units (IU) and 0.1% triamcinolone acetonide 40 mg/mL were mixed in 1 mL of lignocaine hydrochloride and injected intralesionally, followed by photobiomodulation (100 mW for 60 seconds). The third session of the week included only PBM as per the same parameters as the first session. PBM was performed using alternate single wavelength (810 nm) and combo coherence (640 nm and 810 nm at 100 mW each). All sessions are scheduled with a gap of 48 hours. The patient was advised cold soft diet. The patient was prescribed oral antioxidants (capsule Oxitard, twice a day), oral fibrinolytics (tablet colchicine 0.5 mg, once daily), and a multivitamin tablet (SM Fibro, once daily) for three months.

**Figure 4:**
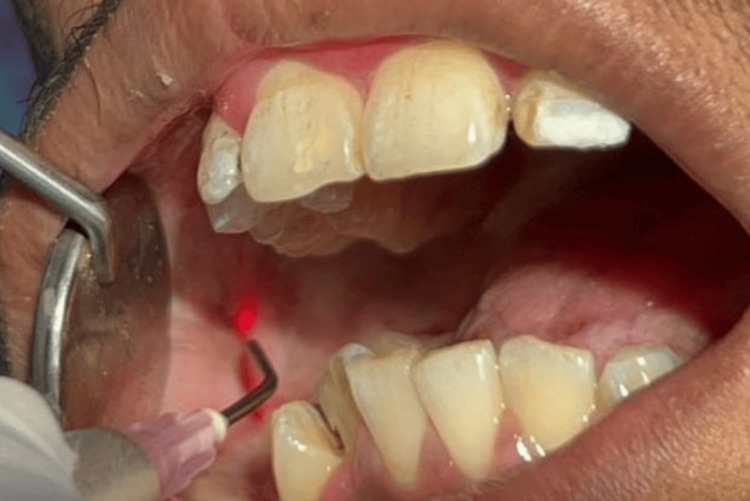
Superficial excision of fibrous bands with a diode laser (NovoLase Gold 810 nm)

By the end of three weeks of sessions, the patient experienced a reduction in burning sensation as measured on a verbal rating scale from eight (preoperative) to four (postoperative). From the fourth week onward, a surgical session was introduced in the third session wherein a minimal superficial cut was made with a diode laser at a power setting of 4000 W, pulsed mode, after local infiltration with 2% lignocaine with adrenaline of 1:80000 dilution. The patient was asked to open his mouth as wide as possible, and the buccal bands were palpated. The areas with thick fibrous bands were then released with a superficial cut. The procedure was done alternatively on the right and left side once a week until the point where no more continuous fibrous bands were felt (Figure 4).

At the end of each surgical session, PBM was performed in combo coherence mode (810+640 nm, 100 mW each for 60 seconds). The whole procedure was repeated for the next two weeks until the end of the total six-week treatment regime.

The patient was also advised to perform various exercises to improve the flexibility and protrusion of the tongue: tongue blade exercise, tongue-in-cheek push, side tongue stretch, cheek puff, pucker, up and down tongue stretch, and teeth sweep. The patient was also advised to perform ice cream stick exercises five times daily for forceful mouth opening [11,12].

Result

**Figure 5:**
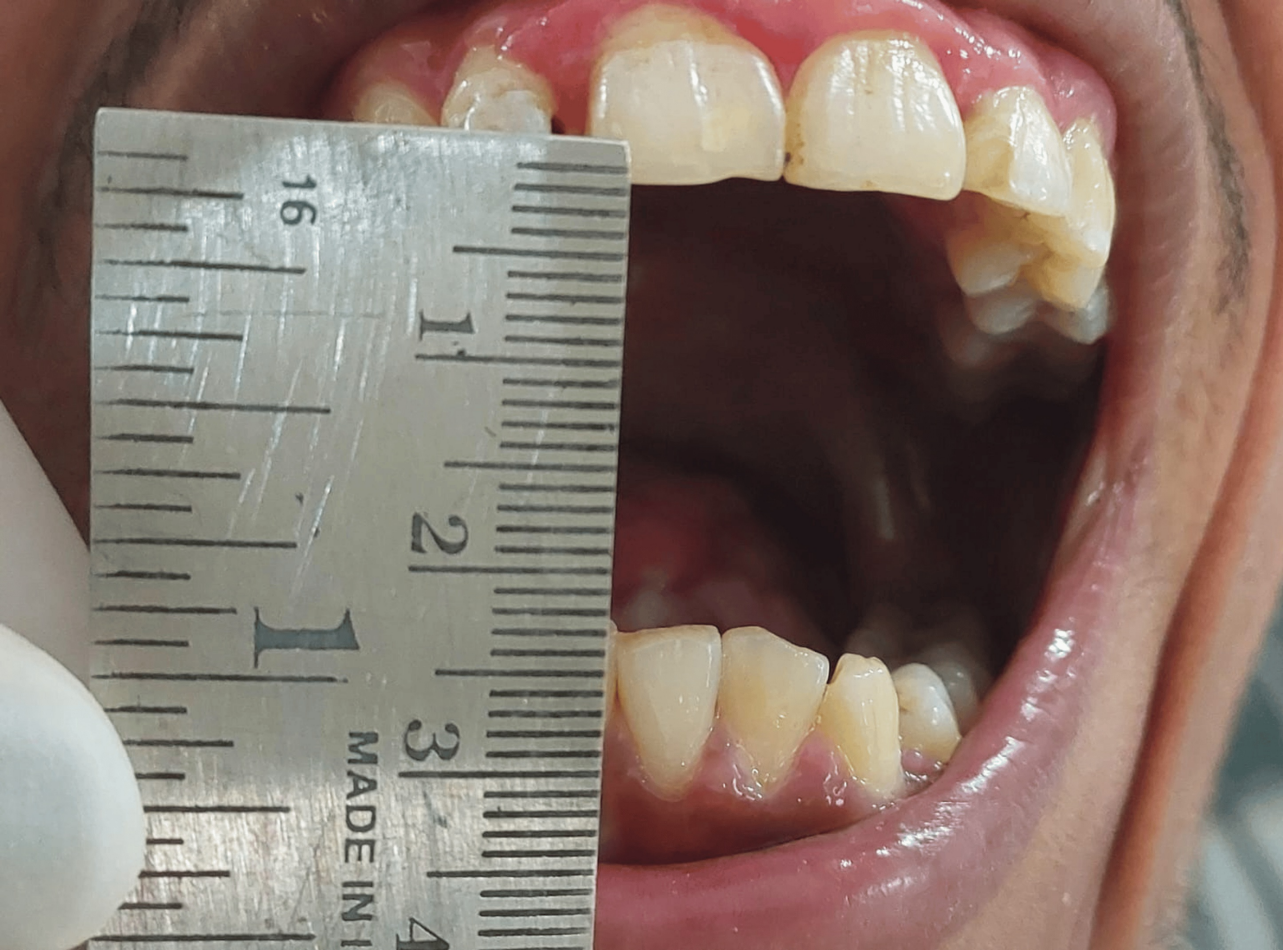
Postoperative inter-incisor distance of 24 mm

At the end of six weeks of treatment, signs of improved mucosa were evident from the earlier stiff and fibrous band of mucosa. There was a gradual improvement in mouth opening with a maximum increase of about 10 mm (first week, 17 mm; second week, 20 mm; third week, 22 mm; fourth week, 22 mm; fifth week, 23 mm; and sixth week, 24 mm; Figure 5).

The burning sensation in the oral cavity was reduced, and the patient was able to eat routine food. The patient gave positive feedback regarding the ability to open the mouth and improvement in speech.

## Discussion

Oral submucous fibrosis has a complex etiology, with areca nuts and tobacco products being the major contributing factors. Other potential causes of OSMF include capsaicin in peppers and chilies, deficiencies in micronutrients such as iron and zinc, and a lack of essential vitamins. The disease may also have an autoimmune origin, as suggested by the presence of specific autoantibodies and associations with certain human leukocyte antigens (HLA). Some research has also indicated genetic factors in the development of the disease [13].

The elasticity of oral mucosa is dependent on the structural integrity of collagen fibers and connective tissue underlying the oral epithelium. The maximum average mouth opening of 18-30 years is 56.60 mm for males and 51.04 mm for females in healthy individuals [14]. Areca nut induces fibrotic pathways by upregulating inflammatory cytokines such as transforming growth factor-beta (TGF-β) and expressing additional cytokines. Moreover, it triggers the conversion of fibroblasts to myofibroblasts, characterized by alpha-smooth muscle actin and gamma-smooth muscle actin (α-SMA and γ-SMA) expression, resulting in accelerated collagen production [1].

Interdisciplinary treatment approaches for OSMF, including both medical and surgical interventions, have been attempted. Since areca nut chewing is a significant etiological factor in OSMF pathogenesis, the primary therapy should focus on habit cessation. The cessation of the habit is adequate to treat at early stages of the disease, but surgery, physical therapy, or combinations of these have been used for moderate-to-severe cases. However, the results of these management methods have been unsatisfactory, expensive, invasive, and sometimes unreliable. Hence, there is a need for novel strategies for the treatment of OSMF. Sheshaprasad et al. reported that the frequency and duration of habit showed no correlation with disease severity, but it is the first milestone of management [15].

Colchicine, an alkaloid known as colchicine-N-acetamide, is an anti-fibrotic agent. Colchicine also has anti-inflammatory properties. These effects are due to its impact on polymorphonuclear leukocytes, monocyte chemotaxis, leukocyte adhesiveness, and prostaglandin E, which suppress leukocyte function. Colchicine inhibits collagen synthesis by interfering with procollagen secretion and its conversion to collagen. It specifically disrupts microtubule formation and inhibits microtubule polymerization by binding to tubulin [16].

Triamcinolone acetonide is a systemic steroid that suppresses the immune system by reducing the activity and volume of the lymphatic system. It heals inflammatory mucosal lesions by decreasing inflammation, suppressing the migration of polymorphonuclear leukocytes, and reversing capillary permeability. It is considered a superior corticosteroid for intralesional injection due to its properties, such as higher local potency, longer duration of action, and reduced systemic absorption.

Hyaluronidase is an enzyme that reduces the viscosity of the ground substance, making tissues more permeable to the injected corticosteroid triamcinolone acetonide. It stimulates the hydrolysis of hyaluronic acid, a key component of tissue cement that resists the diffusion of liquids through tissues. This enzyme facilitates the distribution and absorption of locally injected substances and promotes the resorption of excess fluids and extravasated blood in the tissues. Daga et al. evaluated the effectiveness of oral colchicine with the intralesional injection of hyaluronidase and the injection of triamcinolone acetonide in patients with grade II OSMF. The results showed that the use of intralesional hyaluronidase with oral colchicine resulted in better outcomes, with increased mouth opening and reduction in burning sensation, without significant side effects [16]. Shil et al. reported that colchicine and the intralesional injection of hyaluronidase should be considered preferable to the intralesional injection of steroids [17].

The biostimulatory or inhibitory effects of PBM are governed by the Arndt-Schulz law. The law states that strong stimuli will inhibit physiological activity and low doses will increase the physiological process. The PBM effect can be achieved within an output power typically in the range of 50-500 mW, with wavelengths between 630 and 980 nm. Importantly, PBM achieves its effects on tissues without raising their temperature and has no known adverse effects. PBM acts on cells by interacting with cytochrome C oxidase. This interaction results in an increase in mitochondrial membrane potential upon the absorption of the laser light at an appropriate dose. The anti-inflammatory and analgesic properties post-PBM can be attributed to a reduction of pro-inflammatory cytokines [18].

Therefore, PBM helps in reduced burning sensation, improved healing, accelerated repair, and vascularization as evident in the outcome of our present case. Sukanya et al. concluded that PBM can be used as a dependable treatment modality with considerable improvement in function and quality of life for patients with OSMF [9]. Aparna et al. concluded that immediate results with laser therapy are promising, whereas intralesional steroid injections remain more effective for achieving long-term effects [19].

The conventional surgical management of OSMF with a blade includes incision and the release of bands but has a high recurrence from contracture. Consequently, various surgical grafts have been recommended not only to cover the raw area but also to avoid postsurgical contracture. However, the use of surgical grafts involves the use of extensive surgical procedures associated with their own set of limitations and postoperative complications and discomfort to the patients in certain cases. In this context, a diode laser provides a viable alternative as it does not involve a hospital stay and prolonged follow-up as compared to other surgical interventions. The diode laser with its handpiece also has the advantage of reaching relatively inaccessible areas in patients with restricted mouth opening. With a cutting depth of less than 0.01 mm, controlled cutting is possible with no damage to deeper structures. It seals smaller blood and lymphatic vessels, allowing excellent visibility and minimal chances of postoperative bleeding and edema. Existing evidence reveals that the diode laser has been used in a maximum number of studies to treat OSMF in the form of either laser fibrotomy or the noninvasive nature of PBM. Due to the minimal morbidity associated with this procedure, better patient compliance can be experienced. Also, the procedure can be repeated if required [20]. Despite a few limitations such as high cost, the need for advanced training and safety measures, and delayed wound healing, laser has become indispensable in the management of OSMF. Further long-term studies would be required to assess the suitable laser parameters and the rate of the recurrence of symptoms associated with OSMF.

## Conclusions

Oral submucous fibrosis is a complex, potentially malignant condition that requires an incremental treatment plan. For such complicated conditions, an interdisciplinary approach can help promote more promising outcomes in combination with intralesional injections and home care. A novel approach with minimally invasive laser therapy and PBM can deliver promising results. For the successful long-term effects of laser in OSMF patients, this therapy has to be followed by some postoperative adjunctive aids such as physiotherapy, the cessation of habit, other nutritious supplements, and regular follow-up to evaluate the improvement in oral symptoms. Henceforth, such alternative treatment options would increase the acceptance of treatment for patients suffering from OSMF.
